# Long-term outcomes of venous graft preservation in a rat autologous transplant model

**DOI:** 10.1038/s41598-026-55596-5

**Published:** 2026-06-04

**Authors:** Manuel J. Santander, Aiyana Mardus, George Kensah, Niels Voigt, Bernhard C. Danner, Tomislav Stojanovic, Aschraf El-Essawi, Hassina Baraki, Ingo Kutschka, Ahmad Fawad Jebran

**Affiliations:** 1 Department of Thoracic and Cardiovascular Surgery, University Medical Center Göttingen, Robert Koch Strasse 40, D-37075 Göttingen, Germany; 2 Partner site Göttingen, DZHK (German Centre for Cardiovascular Research), Göttingen, Germany; 3 Department of Cardiac Surgery, University Hospital Basel, Basel, Switzerland; 4 Institute of Pharmacology and Toxicology, University Medical Center Göttingen, Göttingen, Germany; 5 Vascular and Endovascular Surgery Hospital Wolfsburg, Wolfsburg, Germany

**Keywords:** Venous grafts, Preservation solutions, Vascular remodeling, Coronary artery bypass grafting, Rat model, Cardiology, Diseases, Medical research

## Abstract

Venous grafts remain the most used conduits in coronary artery bypass grafting (CABG) but are highly susceptible to early failure due to ischemia–reperfusion injury, intimal hyperplasia, and accelerated atherosclerosis. Specialized preservation solutions such as DuraGraft and TiProtec aim to protect endothelial integrity during intraoperative storage; however, their long-term effects on venous graft adaptation remain unclear. In this study, saline, DuraGraft, and TiProtec were compared in a rat model of arterialized autologous jugular vein interposition grafting. Male Wistar rats were assigned to one of the three storage solutions or a Sham group with immediate implantation. Jugular veins were harvested, preserved for 2 h, and implanted into the infrarenal abdominal aorta using end-to-end microsurgical anastomoses. After 16 weeks, all grafts remained patent and exhibited pronounced arterial remodeling, including dilation, wall thickening, intimal hyperplasia, and loss of endothelium-dependent relaxation, while endothelium-independent relaxation was preserved. Although TiProtec-treated grafts showed slightly enhanced relaxation compared with saline-treated grafts, no preservation solution demonstrated a significant functional or structural advantage at 16 weeks. These findings suggest that long-term arterial loading is the dominant determinant of venous graft remodeling.

## Introduction

Coronary artery bypass grafting (CABG) remains the gold standard for patients with advanced multivessel coronary artery disease, particularly when associated with diabetes mellitus or impaired left ventricular function^1,2^. The long-term success of CABG is critically dependent on the patency of the grafts. Although the internal mammary artery demonstrates excellent durability, the choice of the second conduit remains debated. Current evidence consistently points towards a clinical and angiographic benefit of a second arterial graft compared with venous conduits. Nonetheless, the great saphenous vein remains the most frequently used supplementary conduit owing to its length, accessibility, and ease of harvest^3^. However, venous graft failure occurs in up to 25% of patients within the first year and in 50–60% at 10 years^4–6^. Several pathophysiological processes contribute to vein graft failure, including early thrombosis, intimal hyperplasia, and accelerated atherosclerosis^7–9^. During graft harvest and storage, ischemia–reperfusion injury can trigger endothelial damage and smooth muscle activation, promoting thrombogenicity and maladaptive remodeling^10^. To mitigate this process, specialized preservation solutions have been developed to protect the vascular endothelium and maintain graft viability between harvest and implantation. Historically, normal saline or autologous blood has been used, but both have been shown to impair endothelial integrity^11^. More recently, buffered preservation solutions enriched with antioxidants and amino acids—such as DuraGraft (Marizyme, Jupiter, USA), containing glutathione, L-ascorbic acid, and arginine, and TiProtec (Dr. Franz Köhler Chemie GmbH, Bensheim, Germany), derived from histidine–tryptophan–ketoglutarate solution and supplemented with iron chelators and amino acids—have been introduced with promising ex vivo results^12–14^.

While several ex vivo and short-term in vivo studies mainly in arterial grafts suggested endothelial protection by these solutions, it is unknown whether such early benefits translate into durable functional or structural advantages, particularly in venous grafts, once they are chronically exposed to arterial pressure and flow^15,16^. Vascular remodeling of arterialized venous grafts is a dynamic process, characterized by wall thickening, intimal hyperplasia, and altered vasomotor function^17^. It remains uncertain whether the protective effects of storage solutions result in sustained graft protection after graft implantation.

The present study aimed to compare three preservation strategies - normal saline (NS), DuraGraft, and TiProtec - in a rat model of autologous venous interposition grafting. We hypothesized that DuraGraft and TiProtec would preserve vasomotor function and attenuate intimal hyperplasia and wall thickening compared with saline storage.

## Results

### Animal survival and perioperative data

Thirty-five male rats underwent surgery. Perioperative mortality occurred in 7 animals (20%, 3 NS, 3 Sham, 1 DuraGraft), while all rats in the TiProtec group survived (Table 1). Deaths were mainly attributable to perioperative hemorrhage (Sham, *n* = 1; NS, *n* = 2; DuraGraft, *n* = 1) and intraoperative graft thrombosis (Sham, *n* = 2; NS, *n* = 1), without evidence of group-specific clustering of causes; survival analysis showed no significant between-group difference (log-rank *p* = 0.15). Baseline body weights and operative times were comparable across groups. At 16 weeks, all surviving grafts were patent with palpable pulses and macroscopically open lumina.

**Table 1:** Perioperative data. Data is presented as mean ± standard deviation.

	Sham	Normal saline	DuraGraft	TiProtec
Rats (n)	7	10	9	9
Death (n)	3	3	1	0
Available for analysis (n)	4	7	8	9
Weight (g; mean *±* SD)	354 ± 18	357 ± 28	346 ± 18	351 ± 43
Native jugular vein explantation time (min; mean *±* SD)	14 ± 2	14 ± 2	16 ± 2	18 ± 4
Aortic cross-clamp time (min; mean *±* SD)	25 ± 3	28 ± 3	26 ± 3	29 ± 4

### Vascular contractile response

Myography was successfully performed in 18 explanted grafts (NS *n* = 5, DuraGraft *n* = 5, TiProtec *n* = 5, Sham *n* = 3) and five native jugular veins.

**Fig. 1:**
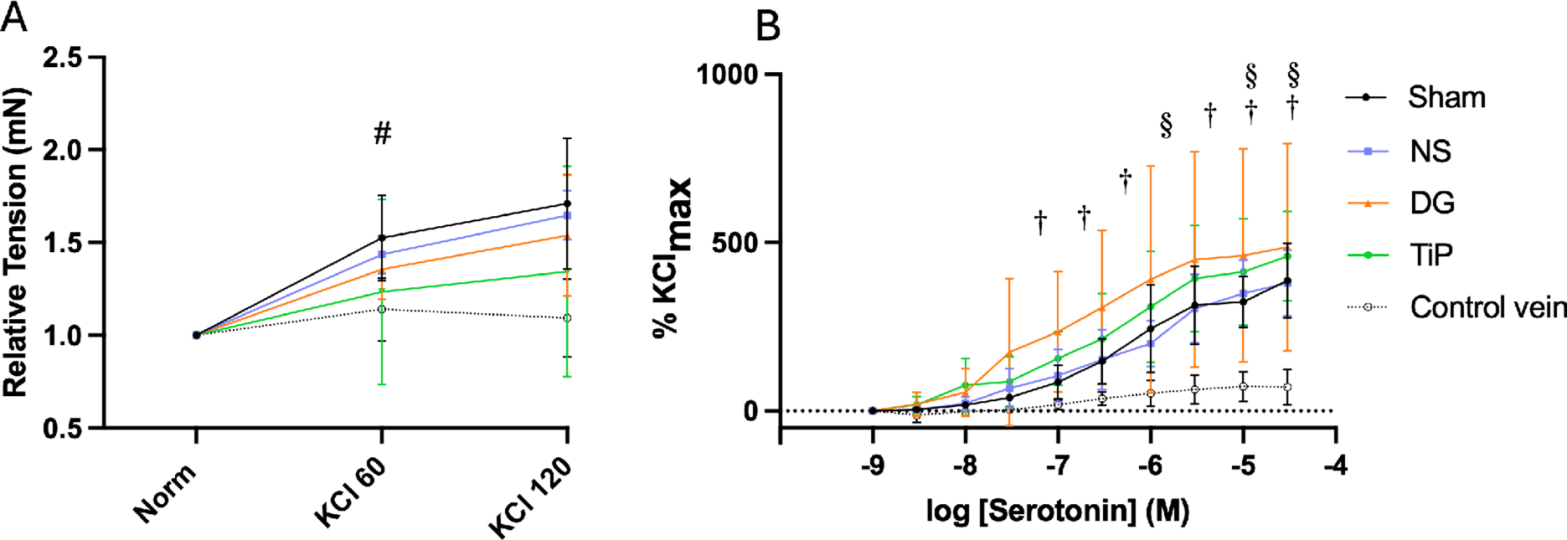
Effect of preservation solutions on contractile responses to potassium chloride and serotonin 16 weeks after vein transplantation. (**A**) Starting from normalisation, stepwise high-potassium (KCl) depolarisation was performed using 60 mM and 120 mM KCl. KCl-induced contractions are expressed as tension development (mN) relative to the normalized resting baseline. (**B**) Serotonin-induced contraction, expressed as a percentage of the maximal response to 120 mM KCl. Data are shown as mean ± SD. # *p* < 0.05: TiP vs. Sham, NS, and DG. §: TiP vs. native control vein. †: DG vs. native control vein. NS, normal saline (*n* = 5); DG, DuraGraft (*n* = 5); TiP, TiProtec (*n* = 5); Sham (*n* = 3); native control vein (*n* = 5). KCl, potassium chloride.

All grafts showed strong receptor-independent contractions in response to high extracellular potassium (120 mM KCl). Tension development among stored grafts was most pronounced in NS and DuraGraft preserved grafts, whereas TiProtec-treated grafts exhibited the lowest responses (Fig. 1A). At 60 mM KCl, one-way ANOVA revealed a significant difference among groups (*p* = 0.002). Post-hoc testing showed that contractility was significantly reduced in the TiProtec group compared with DuraGraft (*p* = 0.019), normal saline (*p* = 0.004), and Sham (*p* = 0.003). No significant differences were detected between groups at 120 mM KCl.

Serotonin exposure elicited marked contractions in all transplanted grafts, consistently exceeding those observed in native jugular veins (Fig. 1B). The Sham group demonstrated response curves comparable to those of the stored grafts. Although grafts after DuraGraft and TiProtec storage showed slightly steeper concentration–response curves, maximal contraction and pD₂ values to serotonin were similar among all graft groups, with no statistically significant intergroup differences (Table 2).

**Table 2:** The impact of different preservation solutions on vein graft function after implantation into the abdominal aorta as assessed by wire myography. Serotonin-induced contractions are presented as percentage of the maximal response to 120 mM potassium chloride. Maximal relaxations to acetylcholine and sodium nitroprusside are expressed as percentage of the serotonin-induced contraction. SNP, Sodium nitroprusside, R_max_ denotes the maximal relaxation, and pD₂ represents the negative logarithm of the half-maximal effective concentration (EC₅₀). Normal saline (*n* = 5); DuraGraft (*n* = 5); TiProtec (*n* = 5); Sham (*n* = 3). Data is presented as mean ± standard deviation.

	Sham	Normal saline	DuraGraft	TiProtec	*P* value
KCl (mN)	2.91± 0.69	3.54 ± 0.58	3.56 ± 1.15	2.29 ± 0.55	0.0967
Serotonin (%)	386 ± 110.40	379.56 ± 97.32	486.05 ± 307.12	459.24 ± 132.73	0.6569
pD_2_ to Serotonin	6.18 ± 0.67	6.04 ± 0.44	6.92 ± 0.72	6.12 ± 0.91	0.1651
R_max_ to Acetylcholine (%)	3.55 ± 7.39	-2.85 ± 6.03	3.64 ± 5.11	3.05 ± 7.49	0.4220
pD_2_ to Acetylcholine	n.d.	n.d.	n.d.	n.d.	n.d.
R_max_ to SNP (%)	61.51 ± 12.28	56.59 ± 9.79	68.16 ± 14.33	75.46 ± 5.29	0.0687
pD_2_ to SNP	6.56 ± 0.53	6.43 ± 0.22	6.74 ± 0.64	6.73 ± 0.49	0.4238

### Endothelium-dependent and endothelium-independent relaxation

**Fig. 2:**
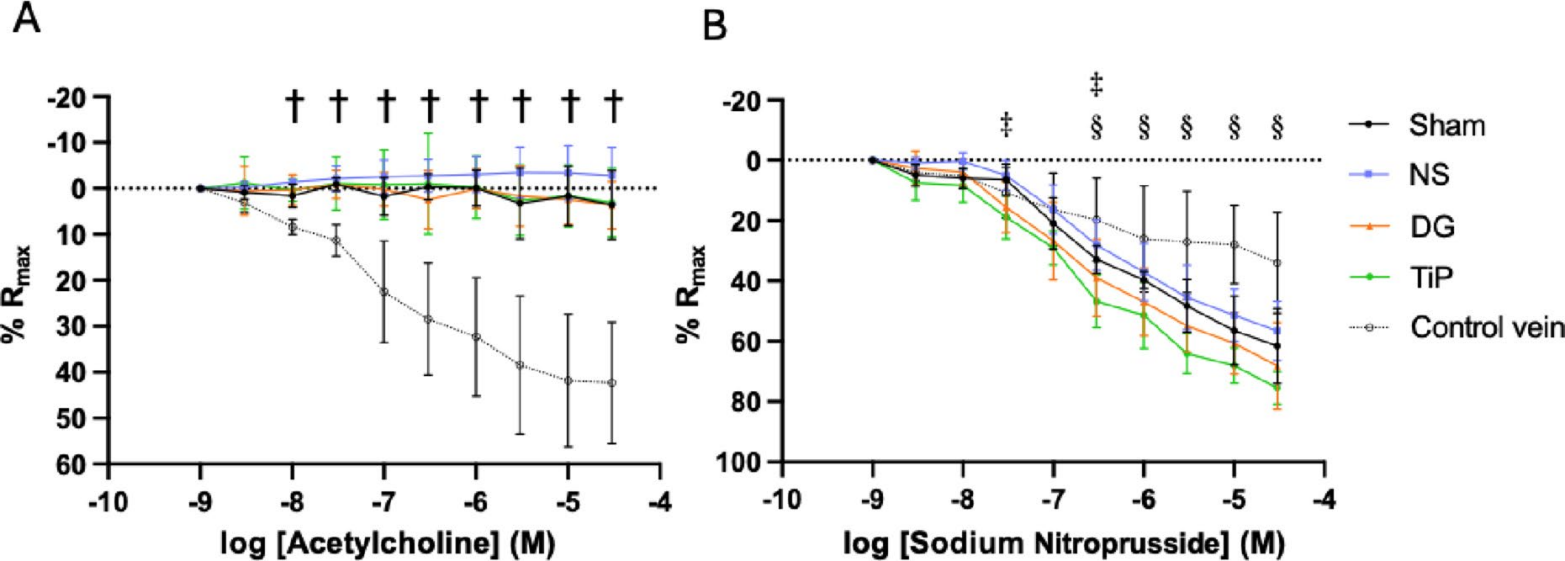
Effect of preservation solutions on vasorelaxation responses 16 weeks after vein transplantation. (**A**) Acetylcholine-induced, endothelium-dependent relaxation and (**B**) sodium nitroprusside-induced, endothelium-independent relaxation in arterialized vein rings. Maximal relaxations to acetylcholine and sodium nitroprusside are expressed as percentage of the serotonin-induced contraction. Data is presented as mean ± SD. *p* < 0.05: † native control vein vs. all graft groups. §: TiP vs. native control vein. ‡: NS vs. TiP. NS, normal saline (*n* = 5); DG, DuraGraft (*n* = 5); TiP, TiProtec (*n* = 5); Sham (*n* = 3); native control vein (*n* = 5).

Endothelium-dependent vasorelaxation with acetylcholine was profoundly impaired in all grafts. None of the graft groups exhibited a discernible concentration–response curve, and pD₂ values could therefore not be determined (Table 2). In contrast, native jugular veins showed a clear, dose-dependent relaxation, reaching highly significant differences at the maximal concentration (*p* < 0.0001 vs. all graft groups; Fig. 2A).

Endothelium-independent relaxation to sodium nitroprusside was preserved across all graft groups and consistently exceeded the responses observed in native veins at higher concentrations (Fig. 2B). TiProtec-treated grafts demonstrated slightly enhanced relaxation compared with NS, reaching significance at the 3 × 10⁻⁷ M concentration (*p* = 0.033). However, maximal relaxation and pD₂ values to sodium nitroprusside were similar among all preservation groups, with no significant intergroup differences (Table 2).

### Histomorphological remodeling of venous grafts

**Fig. 3:**
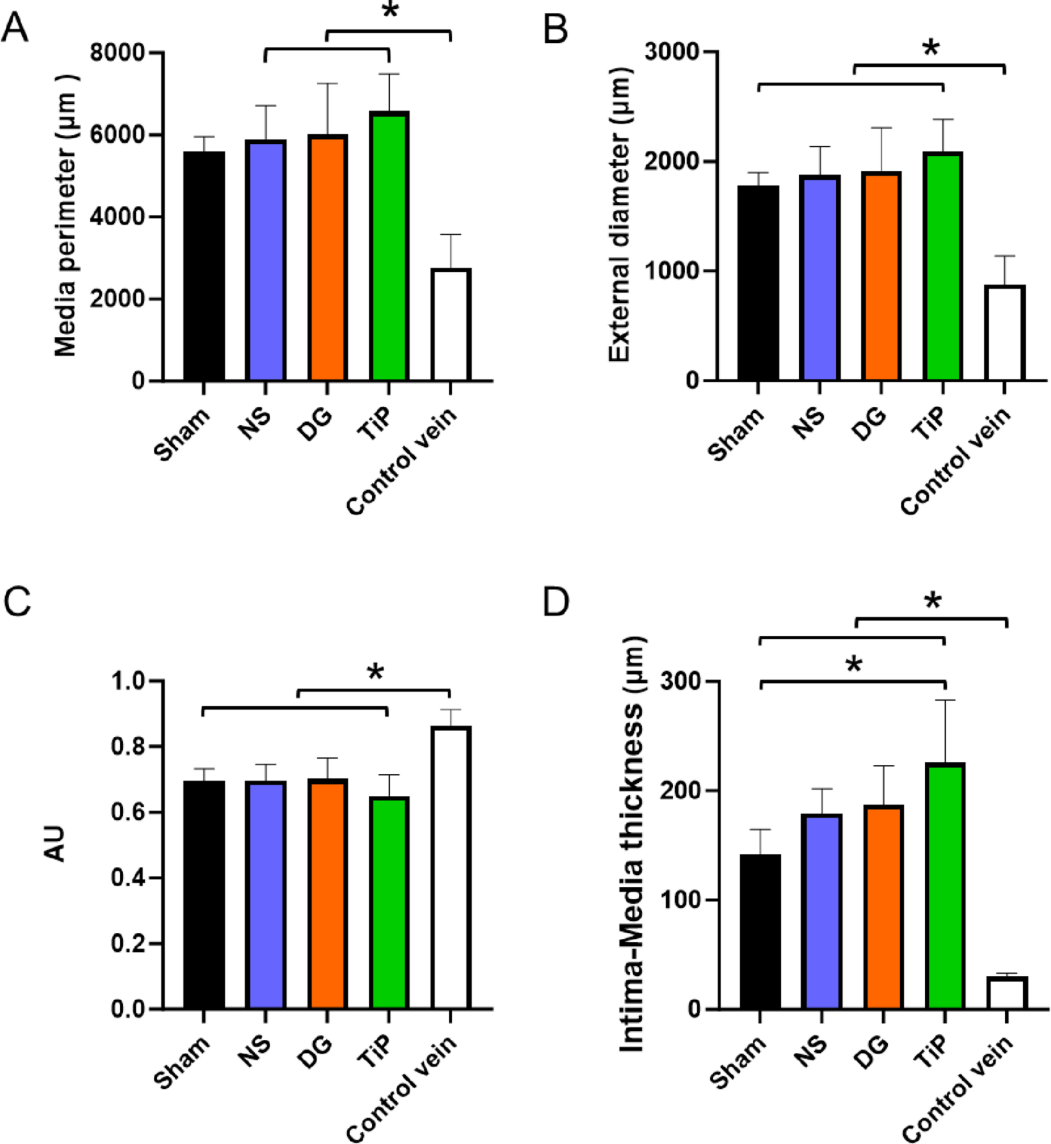
Quantitative histomorphometric analysis of the explanted vein grafts 16 weeks after vein transplantation. (**A**) Media perimeter. (**B**) External diameter of the media. (**C**) Lumen-to-wall ratio. (**D**) Intima-media thickness. Data is presented as mean ± standard deviation. NS, normal saline (*n* = 7); DG, DuraGraft (*n* = 8); TiP, TiProtec (*n* = 9); Sham (*n* = 4); native control vein (*n* = 6). AU, arbitrary units. *p < 0.05.

H&E staining demonstrated pronounced remodeling in all implanted veins, characterized by vessel enlargement, wall thickening, and prominent intimal hyperplasia compared with native jugular veins. All grafts exhibited marked increases in external diameter (one-way ANOVA, *p* < 0.001) and media perimeter (Kruskal–Wallis, *p* = 0.001) relative to native veins (Fig. 3A and B). TiProtec-treated grafts showed the largest diameters, although this difference did not reach statistical significance compared with NS- or DuraGraft-preserved grafts.

Due to the pronounced wall thickening and outward remodeling, the lumen-to-wall ratio was significantly reduced in all grafts compared with native control veins (one-way ANOVA, *p* < 0.001; Fig. 3C). No significant differences were observed between the experimental groups.

Intima-media thickness was significantly increased in all graft groups compared with native veins (one-way ANOVA, *p* < 0.001; Fig. 3D). Among grafts, the TiProtec group displayed the highest mean values, reaching significance when compared with the Sham group (*p* = 0.006), whereas no other intergroup differences were observed.

**Fig. 4:**
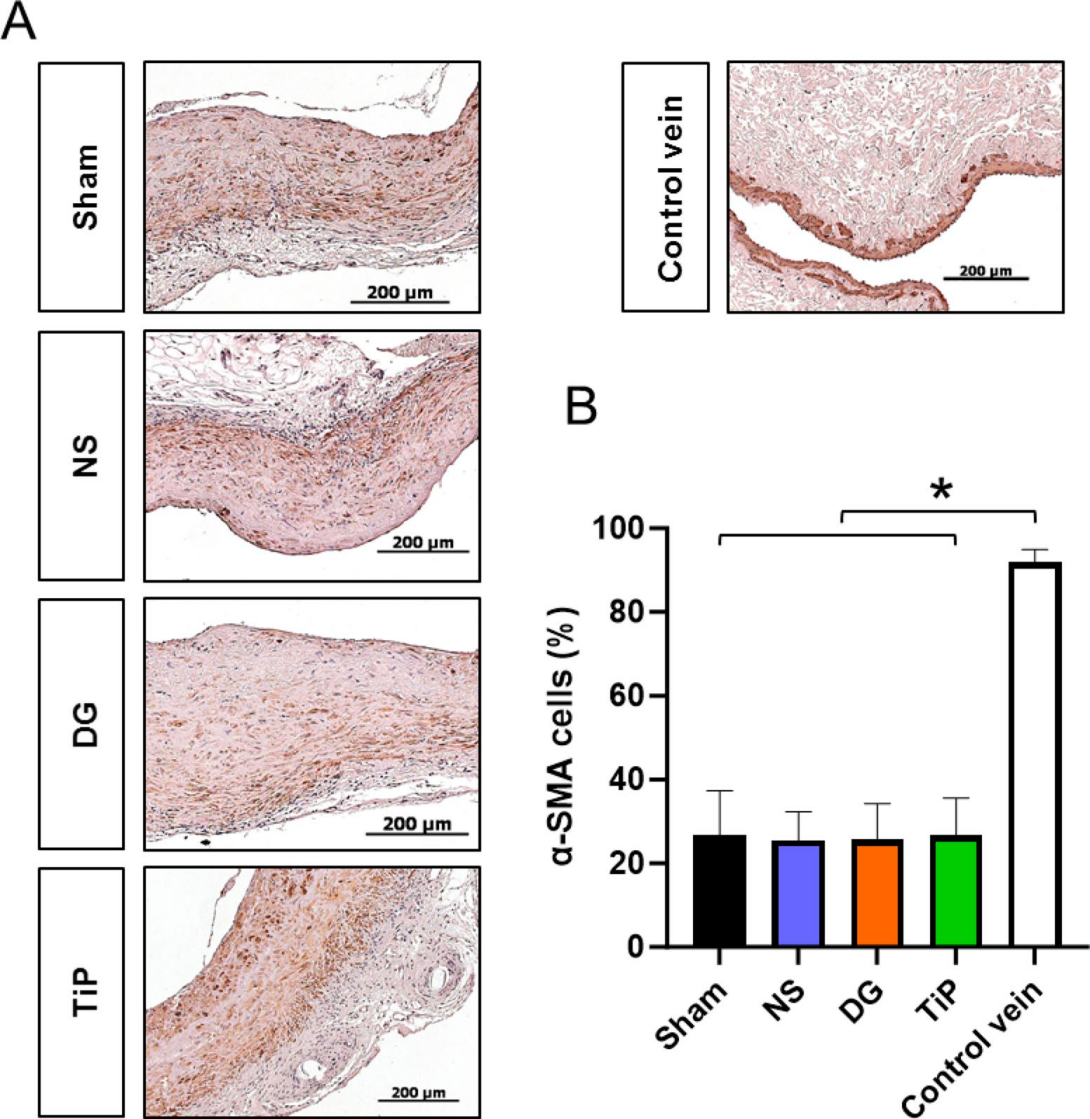
α-Smooth muscle actin staining of venous grafts 16 weeks after vein transplantation. (**A**) Representative micrographs of α-smooth muscle actin (α-SMA) staining (scale bar: 200 μm). (**B**) Quantification of α-SMA–positive cell density across experimental groups. Data is presented as mean ± SD. *p < 0.05: native control vein vs. all graft groups. NS, normal saline (*n* = 7); DG, DuraGraft (*n* = 8); TiP, TiProtec (*n* = 9); Sham (*n* = 4); native control vein (*n* = 6).

Staining against α-smooth muscle actin (α-SMA) demonstrated abundant smooth-muscle–positive cells throughout the neointima and media of all grafts (Fig. 4A). Despite this pronounced smooth muscle presence, quantitative analysis revealed significantly lower α-SMA cell density in all graft groups compared with the native jugular veins (one-way ANOVA, *p* < 0.001; Fig. 4B). Among the preservation groups (NS, DuraGraft, TiProtec) and the Sham group, α-SMA densities were similar, lacking any significant intergroup differences.

## Discussion

This experimental study investigated the long-term effects of three vein graft preservation strategies, saline, DuraGraft, and TiProtec, in a rat model of autologous arterialized jugular vein grafts. After 16 weeks in vivo, all grafts remained patent but demonstrated extensive vascular remodeling, including vessel dilation, wall thickening, intimal hyperplasia, and loss of endothelium-dependent relaxation. However, no significant morphological differences were detected between the three solutions. In the TiProtec group, grafts demonstrated an improved vasomotor function and larger luminal diameters, lacking any statistical significance when compared with the other groups.

Remodeling phenomena as observed in the current study are consistent with findings of prior reports that venous conduits exposed to the arterial circulation undergo profound structural and functional adaptation^18^. Early thrombosis and endothelial injury trigger maladaptive processes that progress to intimal hyperplasia and accelerated atherosclerosis which may lead to graft failure in patients after CABG^7^. The absence of acetylcholine-induced vasodilation in all grafts, even in the Sham group, suggests the early onset of endothelial dysfunction, a hallmark of arterialized veins^15,19^. However, preserved responses to sodium nitroprusside indicate intact smooth muscle cell relaxation capacity, aligning with previous studies in humans and animals^20^. The TiProtec group showed a stronger vasodilatory response at higher doses than the other groups, although this difference was not statistically significant. The beneficial effects of TiProtec as reported by other groups could not be confirmed in our long-term model^13,21,22^.

Specialized storage solutions such as DuraGraft and TiProtec have been shown to preserve endothelial integrity and smooth muscle viability in short-term ex vivo models as well as in clinical observational studies^14,23–26^. However, the present in vivo data suggest that such benefits may be outweighed by the dominant effects of chronic arterial pressure and altered shear stress on venous graft resulting remodeling. The non-significant trend towards improved function in the TiProtec group may reflect partial mitigation of early ischemia–reperfusion injury, although this remains speculative given the absence of early assessment time points. Regardless, this trend did not translate into measurable long-term functional or structural differences after 16 weeks.

A strength of this study is the comprehensive assessment of both functional and structural parameters in an established in vivo model, enabling direct correlation of vascular reactivity with histological remodeling. All microsurgical procedures were performed by a single experienced operator with over ten years of microsurgical expertise. Despite the technical complexity of the jugular vein–to–aorta interposition model, all grafts that survived the perioperative period remained patent at 16 weeks with no late thrombosis, comparing favorably with similar models in which graft thrombosis rates of 22–83% have been reported at 6 weeks^15^. Furthermore, the study design and standardized surgical procedures minimized technical bias.

## Limitations of the study

Several limitations must be acknowledged. First, the use of rat jugular vein as a surrogate for the human saphenous vein introduces species-specific differences in vascular biology^27^. Second, although the 16-week follow-up allowed for assessment of chronic remodelling, preservation solutions are primarily designed to mitigate acute ischemia–reperfusion injury. In a recent aortic transplantation study, DuraGraft preservation attenuated early endothelial dysfunction by reducing inflammatory activation, oxidative stress, and apoptosis^28^. Due to the delayed-assessment design of the present study, such acute protective effects could not be captured. The absence of early time points (e.g., 24 h or 1 week post-implantation) limits our ability to determine whether initial protective effects occurred, and future studies incorporating serial assessments would help clarify whether early graft protection translates into long-term benefits. Third, although overall group sizes were planned a priori based on the primary morphometric endpoint, the number of grafts available for ex vivo wire myography was smaller because functional testing required a sufficiently long and intact proximal ring segment after explantation. As a result, myography could only be performed in a subset of animals, which may have limited statistical power for detection of subtle intergroup differences in vasomotor responses. Fourth, autologous blood was not included as a comparator because of the limited circulating blood volume in rats. Finally, elastin content—a key determinant of arterial wall mechanics and compliance — was not assessed in the present study. Evaluation of elastin fiber remodeling in future studies would provide further insight into the structural adaptation of arterialized venous grafts.

## Conclusions

Our findings emphasize that arterial remodeling dominated in long-term adaptation of arterialized venous grafts overlying any early protective effects of preservation solutions. These results are in accordance with clinical observations that technical and biological factors during and after implantation are major determinants on the outcome of venous graft in CABG^7,8^. Nevertheless, modern preservation solutions such as DuraGraft and TiProtec have the potential of early endothelial protection, which may be of significance during the critical perioperative period.

This knowledge gap may be filled with data from future studies evaluating human venous tissue during early injury as well as during an extended follow-up to assess later phases of atherosclerotic change, which may expand our knowledge on translational relevance.

## Materials and methods

### Animals and ethical approval

All procedures were conducted in accordance with institutional and national regulations for the care and use of laboratory animals and were approved by the Lower Saxony State Office for Consumer Protection and Food Safety (LAVES, Oldenburg, Germany; reference no. 33.9-42502-04-18/2929). Male Wistar rats (8 weeks old) were housed under standard conditions with ad libitum access to food and water and were acclimatized for at least one week prior to experimentation.

### Experimental groups and study design

Eight-week-old male Wistar rats were randomly assigned to four experimental groups: normal saline, DuraGraft, TiProtec and Sham. In the normal saline, DuraGraft and TiProtec groups, the jugular veins were explanted and stored for 120 min at room temperature in the assigned storage solution before implantation. In the Sham group, the veins were explanted and immediately reimplanted without any storage period.

**Fig. 5:**
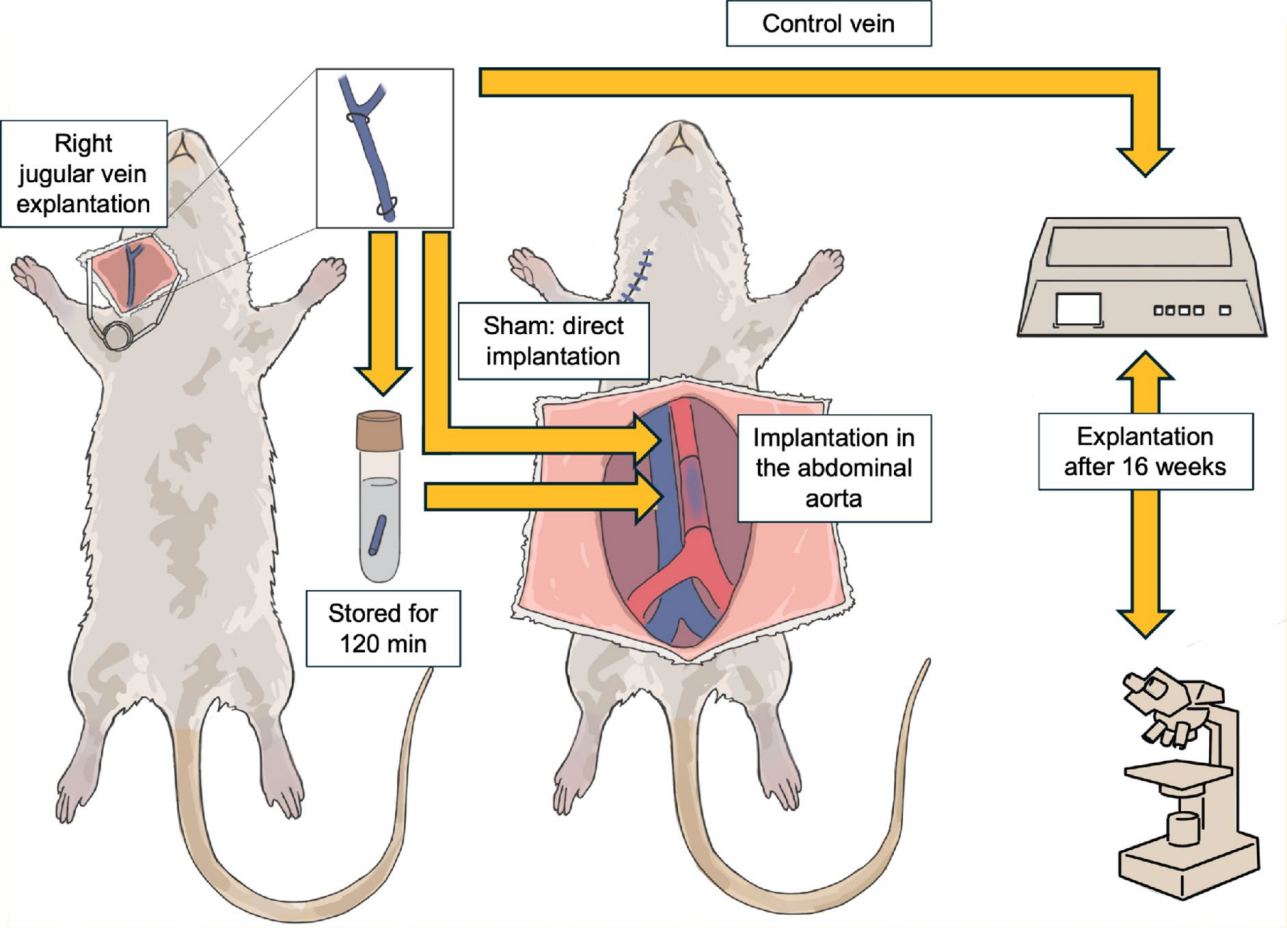
Schematic overview of the animal model and experimental workflow. The right external jugular vein in rats was harvested, followed by either immediate reimplantation (Sham) or storage for 120 min in the assigned preservation solutions. The vein graft was subsequently implanted as an interposition graft into the infrarenal abdominal aorta using interrupted sutures. Grafts were explanted after 16 weeks for vasomotor function testing and histological analyses.

After surgery, all animals were followed for 16 weeks to allow for chronic graft remodeling. At the end of the follow-up period, the grafts were explanted, prepared as vascular rings and mounted in organ baths for ex vivo assessment of vascular function. Adjacent segments were processed for histological and immunohistochemical analyses. A schematic overview of the experimental protocol is provided in Fig. 5.

### Surgical procedure

Surgery was performed under general anesthesia with inhaled sevoflurane and perioperative analgesia (Buprenorphin 0.05 mg·kg⁻¹ s.c; Carprofen 5 mg·kg⁻¹ s.c). The right external jugular vein was exposed via a cervical incision and harvested using microsurgical technique. After preparation, the vein was either immediately reimplanted (Sham) or stored in the assigned preservation solution for 120 min. Except for animals in the Sham group, rats were allowed to recover from anesthesia during the storage period and were re-anesthetized shortly before implantation of the stored vein graft.

For implantation, a midline laparotomy was performed and the infrarenal abdominal aorta was isolated. A 2–3 mm segment of the aorta was excised and replaced with the harvested jugular vein using end-to-end microsurgical anastomoses with 8 − 0 Prolene interrupted sutures under an operating microscope. After hemostasis, the abdomen was closed in layers and animals were allowed to recover under active warming. Postoperative care was provided in accordance with standard veterinary protocols. To prevent thrombosis during surgery and throughout the first three postoperative days, animals received daily subcutaneous injections of unfractionated heparin 1000 IU·kg⁻¹·day⁻¹. No further anticoagulation or antiplatelet therapy was administered beyond postoperative day 3.

Sixteen weeks after implantation, the venous interposition graft was explanted. Anesthesia and preoperative preparation were performed using the same protocol as for the implantation procedure. Following midline laparotomy, the abdominal aorta containing the venous graft was carefully dissected and clamped proximally to the cranial anastomosis and distally to the caudal anastomosis. The graft was then completely excised using sharp microsurgical scissors. After graft explantation, the vascular clamps were released, and animals were euthanized by exsanguination under deep general anesthesia.

### Ex vivo vascular reactivity

After explantation, the proximal 2-mm segment of each graft was immediately transferred to oxygenated Krebs–Henseleit buffer and mounted in a myograph system (DMT 620 M, Danish Myo Technology, Denmark) when graft length permitted. The Krebs–Henseleit buffer contained (in mM): NaCl 116.1, KCl 5.0, MgCl₂ 1.2, Na₂SO₄ 1.2, NaH₂PO₄ 1.8, NaHCO₃ 20.2, glucose 10.0, and CaCl₂ 2.5 (pH 7.4). Grafts of insufficient length for myography and morphological analysis were used solely for histological examination. The buffer was maintained at 37 °C and continuously gassed with carbogen (95% O₂ /5% CO₂). After meticulous removal of surrounding tissue, the rings were mounted on hooks, normalized to their optimal resting tension according to the manufacturer’s guidelines, and allowed to equilibrate.

Following normalization, cumulative concentration–response curves were constructed to assess vascular reactivity. Receptor-independent contraction was evaluated by exposing the rings to two concentrations of potassium chloride (60 and 120 mM), whereas receptor-dependent contraction was assessed using cumulative concentrations of serotonin (10⁻⁹ to 3 × 10⁻⁵ M). Endothelium-dependent relaxation was examined by adding increasing concentrations of acetylcholine (10⁻⁹ to 3 × 10⁻⁵ M) to rings precontracted with serotonin, and endothelium-independent relaxation was assessed in an analogous manner using sodium nitroprusside (10⁻⁹ to 3 × 10⁻⁵ M).

Changes in isometric tension were continuously recorded, and contractile responses were expressed relative to the maximal KCl-induced contraction. Relaxation responses were expressed as a percentage of the preceding serotonin-induced contraction. For each curve, maximal response (E_max_ / R_max_) and vascular sensitivity were obtained by fitting individual concentration–response curves to a four-parameter logistic equation:

$$\mathrm{Y}=\mathrm{Bottom}+\frac{\left(\mathrm{Top}-\mathrm{Bottom}\right)}{1+{10}^{\left(\right(\mathrm{log}\mathrm{EC}_{50}-\mathrm{X}\left)*\mathrm{HillSlope}\right))}}$$

where Top and Bottom denote the upper and lower response plateaus, EC₅₀ the half-maximal effective concentration, X the log molar agonist concentration, and HillSlope the slope factor. Curve fitting was performed using nonlinear regression in GraphPad Prism v10 (GraphPad Software, Boston, MA, USA). The pD₂ was calculated as − log₁₀(EC₅₀).

### Histology and immunohistochemistry

**Fig. 6:**
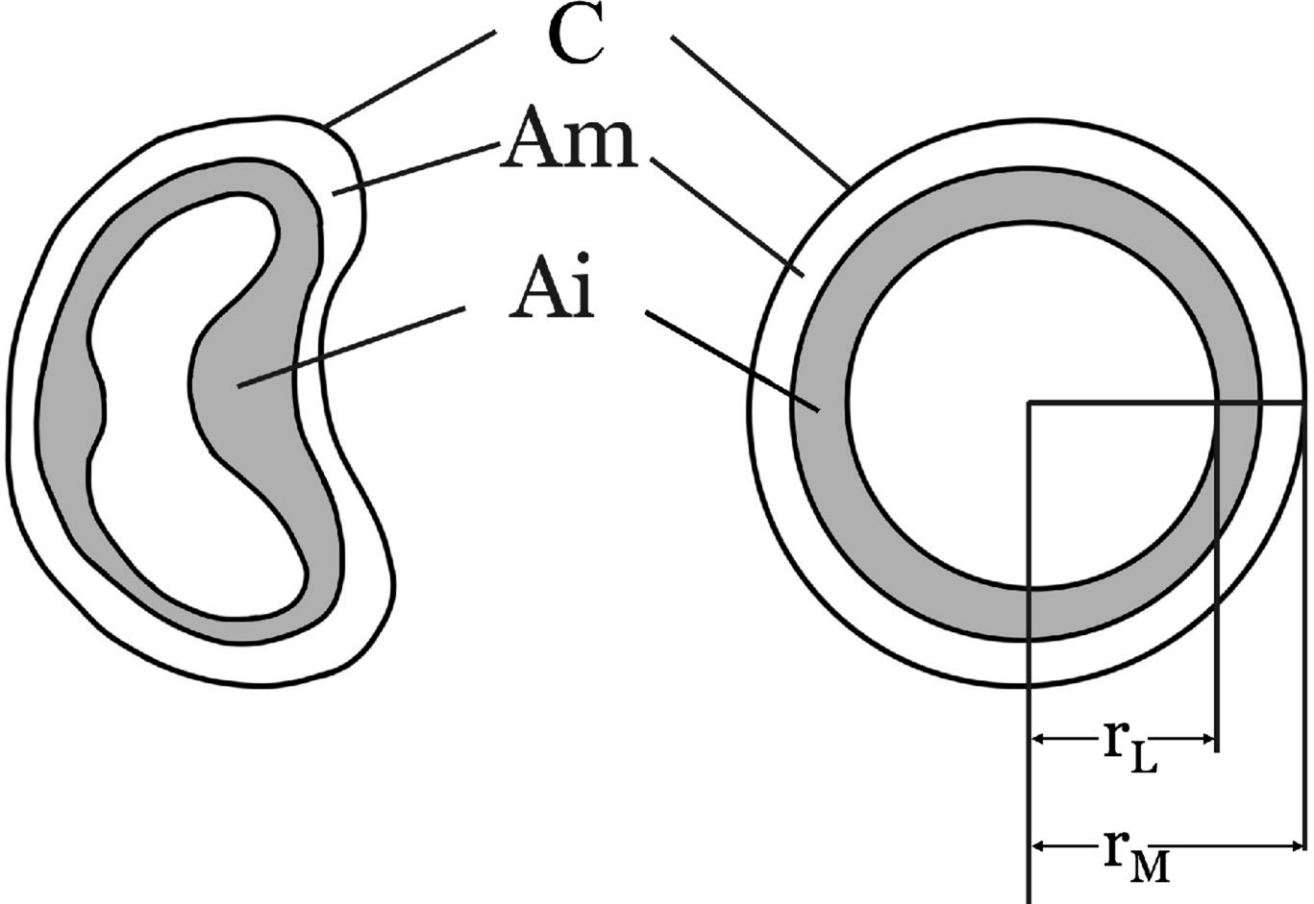
Method for restoring deformed vessels to their original geometry. Vessel diameter was calculated from the external circumference of the tunica media (A_i_, intimal area; Aₘ, medial area; r_L_, luminal radius; r_M_, external medial radius; C, external circumference of the tunica media).

The distal graft segments were fixed in 4% formalin for 24–48 h, embedded in paraffin, and sectioned at a thickness of 4 μm using a rotary microtome. Hematoxylin–eosin staining was performed for morphometric assessment. Sections were examined by light microscopy (BH, Olympus, Tokyo, Japan) coupled to a digital camera and a computer-based image analysis system. Quantitative measurements included lumen diameter, external circumference, wall thickness, and the intima-media area ratio. To calculate vessel circumference, diameter, and luminal area, the method described by Yamaki et al. (1976) was applied to restore deformed vessels to their original shape^29,30^(Fig. 6). From each sample, four regions spaced 90° apart were photographed, as previously described by Navarrete et al.^31^. Morphometric analyses were performed by investigators blinded to the experimental groups using ImageJ software (National Institutes of Health, USA).

In addition, immunohistochemical analysis was performed to assess structural alterations of the vascular media and smooth muscle cells. Sections were incubated overnight at 4 °C with a primary antibody against α-smooth muscle actin (α-SMA; 1:2500), followed by incubation with a rabbit anti-mouse secondary antibody (1:200). Immunoreactivity was visualized using 3,3′-diaminobenzidine (DAB) as chromogen. After washing, sections were counterstained with hematoxylin, dehydrated, mounted, and examined by light microscopy.

### Statistical analysis

Sample size was calculated a priori in 2018, before submission of the animal study protocol, using G*Power (version 3.1.9.2; Heinrich-Heine-Universität Düsseldorf, Germany), as required by the responsible regulatory authority (Lower Saxony State Office for Consumer Protection and Food Safety, LAVES) for approval of the animal experiments. The calculation was based on a one-way ANOVA (fixed effects, omnibus, four groups) using intimal hyperplasia as the primary outcome variable. Assuming a biologically relevant difference of 3500 μm², a standard deviation of 2000 μm², α = 0.0125, and a power of 80%, the required total sample size was 32 animals, corresponding to 8 animals per group. Three additional animals were included to account for potential perioperative losses. To prevent confounding by operator training effects, surgeries were scheduled in a cyclic sequence across all experimental groups rather than performing them consecutively within a group. Animals were operated in the order 1–2–3–4 repeatedly, distributing any potential learning-curve improvements evenly across groups and reducing systematic bias attributable to increasing surgical proficiency.

Data is presented as mean ± standard deviation (SD). Normality was assessed with the Shapiro–Wilk test. Group comparisons were performed using one-way ANOVA with Tukey’s post-hoc test or, when appropriate, the Kruskal–Wallis test applying Dunn’s correction. Post-hoc pairwise comparisons were performed only when the omnibus test reached statistical significance. Survival differences were assessed using Kaplan–Meier analysis with log-rank testing. A *p* < 0.05 was considered statistically significant. Statistical analyses were conducted using GraphPad Prism v10.

## Data Availability

The datasets generated and analysed during the present study are available from the corresponding author upon reasonable request.
